# Comparison of the predictive value of two international guidelines for safe discharge of patients with mild traumatic brain injuries and associated intracranial pathology

**DOI:** 10.1007/s00068-021-01842-6

**Published:** 2021-12-03

**Authors:** Sebastian Vestlund, Sebastian Tryggmo, Tomas Vedin, Per-Anders Larsson, Marcus Edelhamre

**Affiliations:** 1 Department of Clinical Sciences, Faculty of Medicine, Lund University, Lund, Sweden; 2 Research and Development Center, Skaraborg Hospital, Skövde, Sweden

**Keywords:** Intracranial hemorrhage, Traumatic, Skull fractures, Brain injuries, Traumatic, Practice guideline

## Abstract

**Purpose:**

To determine and compare the sensitivity, specificity, and proportion of patients eligible for discharge by the Brain Injury Guidelines and the Mild TBI Risk Score in patients with mild traumatic brain injury and concomitant intracranial injury.

**Methods:**

Retrospective review of the medical records of adult patients with traumatic intracranial injuries and an initial Glasgow Coma Scale score of 14–15, who sought care at Helsingborg Hospital between 2014/01/01 and 2019/12/31. Both guidelines were theoretically applied. The sensitivity, specificity, and percentage of the cohort that theoretically could have been discharged by either guideline were calculated. The outcome was defined as death, in-hospital intervention, admission to the intensive care unit, requiring emergency intubation due to intracranial injury, decreased consciousness, or seizure within 30 days of presentation.

**Results:**

Of the 538 patients included, 8 (1.5%) and 10 (1.9%) were eligible for discharge according to the Brain Injury Guidelines and the Mild TBI Risk Score, respectively. Both guidelines had a sensitivity of 100%. The Brain Injury Guidelines had a specificity of 2.3% and the Mild TBI Risk Score had a specificity of 2.9%.

**Conclusion:**

There was no difference between the two guidelines in sensitivity, specificity, or proportion of the cohort eligible for discharge. Specificity and proportion of cohort eligible for discharge were lower than each guideline’s original study. At present, neither guideline can be recommended for implementation in the current or similar settings.

**Supplementary Information:**

The online version contains supplementary material available at 10.1007/s00068-021-01842-6.

## Background

Traumatic brain injuries are common in all parts of the world [1]. Incidences in Europe vary between 47.3 and 849 per 100,000 of the population, according to a systematic review by Brazinova et al. [2]. These rates are similar and relatively constant when compared with those of an earlier systematic review on the same subject by Tagliaferri et al. [3]. These rates may be even higher at 1,012 per 100,000 of the population, based on estimations by Dewan et al. [1].

The vast majority of traumatic brain injuries can be classified as mild [1, 4]. Mild traumatic brain injury (mTBI) is not universally defined but is most frequently defined as a Glasgow Coma Scale (GCS) score of 13–15 upon presentation [5]. Roughly, 6.3–11% of the patients who suffer mTBI will have an intracranial injury (ICI), such as an intracranial hemorrhage or a fracture of the neurocranium, showing on their computerized tomography (CT) scan of the head. Of these patients, around 3.5% will require neurosurgery, and 1.4% will die [6–8]. The current management of patients with ICIs in many institutions includes a neurosurgical consultation, hospital admission for observation, and possibly a repeat CT [9, 10].

**Fig. 1:**
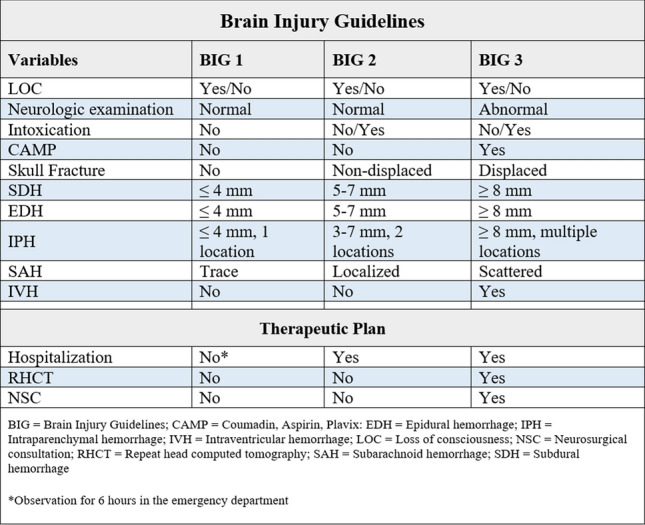
Parameters included in the BIG, along with suggested management plan for each BIG category

However, this management strategy has been questioned in recent years, and studies have investigated whether there is a subset of mTBI patients with ICIs that can be managed without a neurosurgical consultation, a repeat head CT, and a hospital admission [11, 12]. Published by Joseph et al., the brain injury guidelines (BIG) provides a tool for determining in which patients an admission can safely be omitted based on clinical findings, pre-existing medical conditions, and the result of a head CT [13]. In the original study, 9.8% of admissions could have been safely omitted if the BIG were applied. A prospective validation by the BIG’s authors showed that mTBI patients with minor injuries could be managed safely by non-neurosurgeons without an admission, a repeat CT, and a neurosurgical consultation [14]. The parameters included in the BIG can be seen in Fig. 1, along with criteria for each category.

**Fig. 2:**
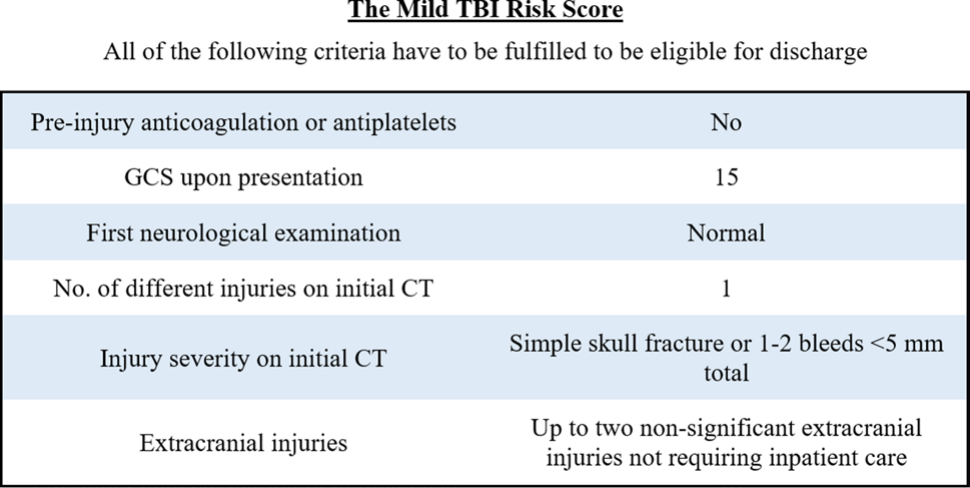
Parameters included in the mTBI RS

In 2020, Marincowitz et al. developed a similar guideline in the UK, named the Mild TBI Risk Score (mTBI RS) [15]. In this study, 5.5% of mTBI patients with ICIs could potentially have been discharged. The authors also tested the BIG criteria for discharge, which in this cohort would lead to 3.6% patients being discharged. Marincowitz et al. speculated that the difference between the theoretical discharge rates of the BIG in their study compared with the original study could be attributed to differences in the study population. The parameters included in the mTBI RS can be seen in Fig. 2.

Both guidelines aim to evaluate the same characteristic—which patients with an ICI can safely be discharged instead of being admitted—but neither of them has received widespread application. Neither one has been tested in a Scandinavian cohort, and before attempting to introduce either of them for clinical use, this needs to be done.

We hypothesized that both guidelines would identify a group of patients with ICIs that would be eligible for safe discharge from the emergency department. We also hypothesized that these proportions would not be significantly different from those in their respective original study. If these hypotheses were shown to be correct, implementing either one of these guidelines could lead to more appropriate utilization of hospital beds and decrease healthcare expenditures.

## Aim

To estimate and compare the sensitivity, specificity, and the proportion of patients eligible for discharge by either the BIG or the mTBI RS in patients with mTBI and concomitant ICI.

## Methods

The study was conducted as a retrospective review of medical records of adult (≥ 18 years) patients with at least one CT-verified ICI and an initial GCS score of 14–15 who presented at Helsingborg Hospital, Sweden, between 2014/01/01 and 2019/12/31.

The population was comprised by patients in northwestern Scania County, with approximately 300,000 inhabitants. The county has both urban and rural areas. The patients were identified through a systemwide search in the electronic medical records for specific ICD-10 codes denoting ICI registered at any point during the hospital admission (Supplementary material 1). After the search, the following exclusion criteria were applied:

- Wrongfully classified as a traumatic injury or no ICI present,
- Revisit for an injury already included in the database
- Patient of a hospital outside Scania County
- Initial management outside Scania County
- Patient registered as deceased at an unknown date after the last registered care event
- Missing initial CT report
- Penetrating injury

To be eligible for guideline comparison, the patients also had to meet the following criteria:

- Level of consciousness can reliably be interpreted
- GCS 14–15

**Fig. 3:**
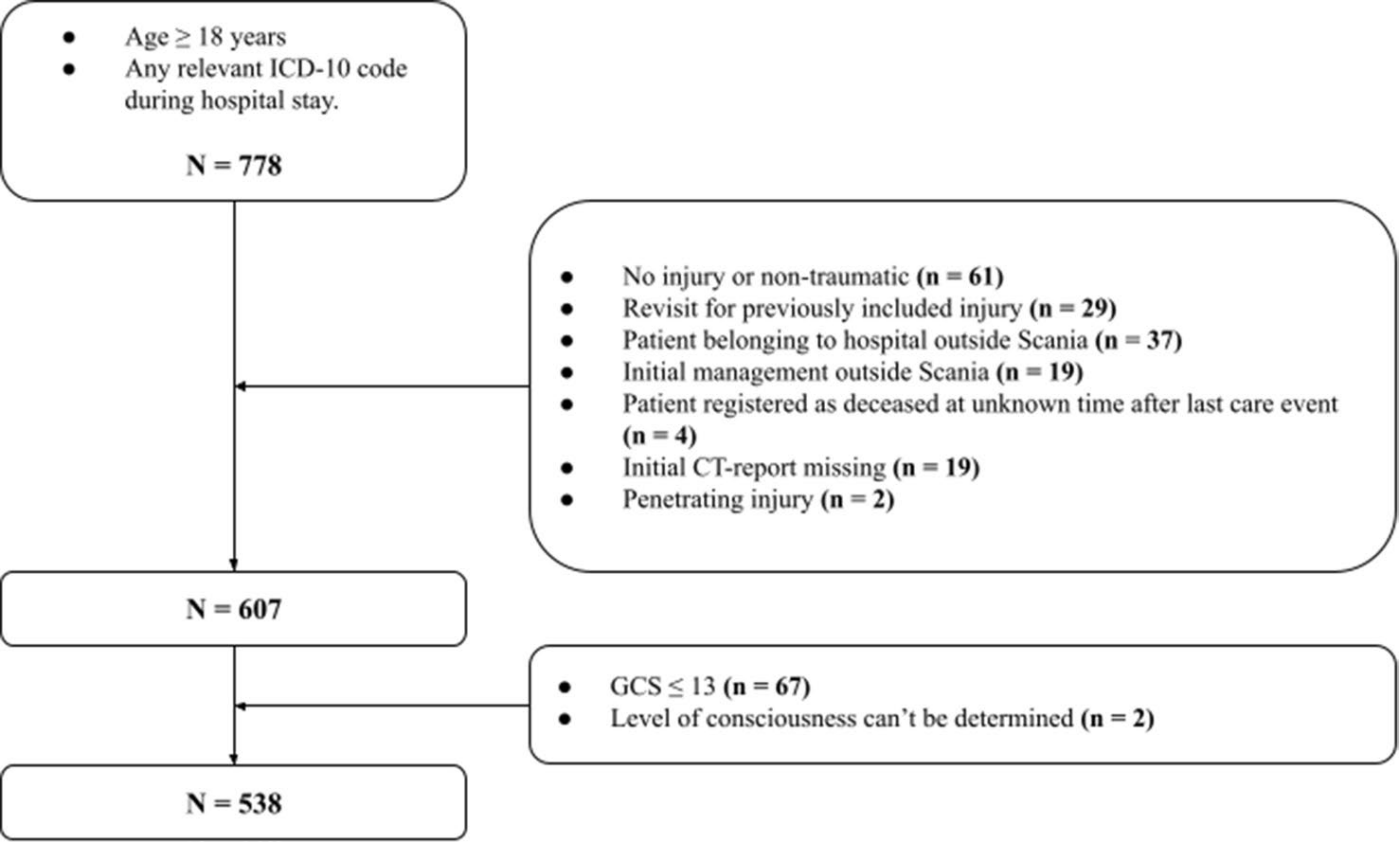
Stepwise exclusion process

In total, 778 patients were identified, of whom 607 remained after the exclusion criteria were applied. The additional exclusion in the second step yielded 538 patients eligible for comparative analysis of the BIG and the mTBI RS. This stepwise exclusion process is shown in Fig. 3.

Prior to the data collection, a control document outlining what data to extract and how to interpret medical records was made (Supplementary material 2). The data were manually extracted from each patient’s medical record by a single interpreter and entered digitally into Microsoft Excel^®^.

Prior to the analysis, the following assumptions and adjustments were made:

- Not mentioning a certain parameter intended for analysis (e.g., loss of consciousness) was interpreted as the absence of that parameter. Equivocal reports were interpreted as positive (e.g., uncertainty regarding loss of consciousness was interpreted as loss of consciousness).
- The GCS was converted from the Reaction Level Scale (RLS), which is the grading scale used in the institution of this study’s researchers [16]. The RLS is inversely converted to the GCS (RLS 1 = GCS 15, RLS 8 = GCS 3), with the exception of RLS 3, which constitutes the range GCS 9–13. In the cases where the RLS was unspecified, it was interpreted from the text in the first note from the emergency department.
- If no comment was made regarding the size of the largest hemorrhage, the radiology report was interpreted by the data collector to identify the hemorrhages that would very likely measure under 5 mm (e.g., “minimal sub-arachnoid hemorrhage” or “punctate parenchymal hemorrhage”).

The data were analyzed using IBM SPSS^®^ Statistics 25. Using the original variables, new variables were constructed to determine which patients would be eligible for discharge based on either the BIG or the mTBI RS. In the patients deemed eligible for discharge based on either guideline, an additional review of the radiology report was conducted to double check the measurements and to rule out cerebellar or intraventricular hemorrhage, since this was not specifically investigated during the primary review. Sensitivity, specificity, and proportion of patients eligible for discharge was calculated for both guidelines when the final eligibility was determined. The two proportions of patients eligible for discharge were compared to each other and to proportions from previous studies using Pearson’s *χ*^2^-test. To evaluate the impact of excluding patients from the RLS 3 category (which includes GCS 13), a second analysis that included the patients with RLS 3 was performed and compared with the original analysis. A *P*-value below 0.05 was considered statistically significant and confidence intervals were set at 95%.

The outcome was deterioration within 30 days of initial presentation. Deterioration was defined as death, requiring in-hospital intervention, admission to the intensive care unit (ICU), emergency intubation due to an ICI, decreased consciousness, or seizure within 30 days of initial presentation. In-hospital intervention was defined as craniotomy, burr hole, placement of an intracranial pressure monitor, administration of mannitol/hypertonic saline to reduce intracranial pressure, or administration of intravenous antibiotics for a basal skull fracture.

Sensitivity was defined as the percentage of patients who deteriorated within 30 days that would have been correctly admitted. Specificity was defined as percentage of patients that did not deteriorate within 30 days that would have been correctly discharged. Eligibility for discharge according to BIG was defined as not fulfilling any criteria for BIG 2 or BIG 3 (thus being BIG 1) and eligibility for discharge according to mTBI RS was defined as fulfilling all criteria presented in Fig. 2.

## Results

The patient characteristics are listed in Table 1 along with corresponding numbers from respective guideline’s original study.

**Table 1:** Patient characteristics and corresponding available data from respective guideline’s original study

Parameter	Category	*N* (*N* = 538)	Missing (%)	BIG [13]	mTBI RS [15]
				(*N* = 1232)	(*N* = 1699)
Age			n/a		
	Median (Q_1_–Q_3_)	76 (63–85)		n/a	n/a
	Mean (SD)	71.5 (18.3)		37.6 (24.6)^a^	58.2 (23.3)
Sex			n/a		
	Female (%)	216 (40.1)		415 (33.7)^a^	560 (33)^a^
	Male (%)	322 (59.9)		817 (66.3)^a^	1139 (67)^a^
Days admitted	Median (Q_1_–Q_3_)	4 (2–9)	n/a	n/a	n/a
RCFS			5 (0.9)		
	Under 50 (%)	58 (10.9)		n/a	649 (39)
	1–3 (%)	267 (50.1)		n/a	642 (38)
	4–6 (%)	183 (34.3)		n/a	308 (18.5)
	7–9 (%)	25 (4.7)		n/a	72 (4.5)
LOC			188 (34.9)		
	Yes (%)	134 (38.3)		756 (61.3)^a^	n/a
	Unsure (%)	111 (31.7)		n/a	n/a
	No (%)	105 (30)		476 (38.7)	n/a
Seizure^b^	Yes (%)	11 (2)	520 (96.7)	n/a	74 (4)
Vomiting^b^	Yes (%)	78 (14.5)	432 (80.3)	n/a	310 (18)
Mechanism of injury			14 (2.6)		
	Ground-level fall (%)	370 (70.6)		n/a	729 (43)
	Elevated fall (%)	67 (12.8)		n/a	361 (21)
	Traffic accident (%)	54 (10.3)		n/a	298 (18)
	Assault (%)	20 (3.8)		n/a	228 (13)
	Other (%)	13 (2.5)		n/a	51 (3)
Documented RLS level	Yes (%)	321 (59.7)	n/a	n/a	n/a
GCS^c^			n/a		
	15 (%)	435 (80.9)		n/a	976 (58)
	14 (%)	103 (19.1)		n/a	533 (31)
Blood pressure	Mean MAP mm Hg (SD), Min–Max	107.3 (18.3), 37–175	8 (1.5)	n/a	98 (17), 43–193
Oxygen saturation	Mean % (SD), Min–Max	96.5 (2.8), 75–100	2 (0.4)	n/a	97.4 (2.4), 80–100
Respiratory rate	Mean Breaths/min (SD), Min–Max	19.1 (4.2), 10–42	8 (1.5)	n/a	17.9 (3.5), 10–48
Hemoglobin	Mean g/L (SD), Min–Max	134.9 (18.8), 69–191	32 (5.9)	n/a	136 (19.1), 68–265
Platelet value	Mean 10^9^/L (SD), Min–Max	236.3 (85.6), 5–852	98 (18.2)	n/a	232 (77), 2–742
Intracranial injury^d^			n/a		
	aSDH (%)	281 (52.2)		519 (42.1)^a^	694 (41)
	SAH (%)	234 (43.5)		606 (49.2)^a^	536 (32)
	IPH (%)	138 (25.7)		461 (37.4)^a^	240 (14)
	naSDH (%)	79 (14.7)			n/a
	EDH (%)	23 (4.3)		110 (8.9)^a^	135 (8)
	Location not specified (%)	38 (7.1)		n/a	n/a
	Other, DAI and edema (%)	17 (3.2)		85 (6.9)^a, e^	n/a
	Linear skull fracture (%)	59 (11.0)		n/a	n/a
	Comminute skull fracture (%)	7 (1.3)		n/a	n/a
	Basilar skull fracture (%)	76 (14.1)		n/a	n/a
Deterioration within 30 days^d^	Total *N* (%)	188 (34.9)	n/a	n/a	470 (27.7)
	Death (%)	37 (6.9)		n/a	72 (4.2)
	In-hospital intervention (%)	114 (21.2)		159 (13)^f^	n/a
	ICU admission (%)	82 (15.2)		n/a	n/a
	Intubation (%)	22 (4.1)		n/a	n/a
	Decreased consciousness (%)	94 (17.5)		n/a	n/a
	Seizure (%)	34 (6.3)		n/a	n/a

*aSDH* acute subdural hematoma, *BIG* brain injury guidelines, *DAI* diffuse axonal injury, *ED* Emergency Department, *EDH* epidural hematoma, *GCS* Glasgow Coma Scale, *ICU* intensive care unit, *IPH* Intra parenchymal hematoma, *IQR* interquartile range, *LOC* loss of consciousness, *MAP* mean arterial pressure, *mTBI* *RS* Mild TBI Risk Score, *N* number, *naSDH* non-acute subdural hematoma, *RCFS* Rockwood Clinical Frailty Scale, *RLS* Reaction Level Scale, *SAH* sub-arachnoid hemorrhage

^a^Estimated using percentages and cohort size from the original article

^b^Prehospital or in the ED. Percentages are presented under the assumption that missing data equated not present

^c^Converted from RLS

^d^Some patients had more than one injury/type of deterioration

^e^Intraventricular hemorrhages

^e^Craniotomy/craniectomy or placement of extraventricular drain

Of the 538 patients, 10 and 11 were eligible for discharge according to the BIG and the mTBI RS, respectively. Five patients had missing data regarding the initial neurological examination, making them all ineligible for the BIG and one for the mTBI RS. An additional review of these subgroups’ initial CT reports identified 1 patient with intraventricular hemorrhage and 1 patient with cerebellar and intraventricular hemorrhage, reducing the BIG group to 8 patients (1.5%) and the mTBI RS group to 10 patients (1.9%) (*p* = 0.63). None of the patients deteriorated within 30 days of admission. The parameters used to determine whether a patient was eligible for discharge according to either the BIG or the mTBI RS are presented in Table 2 along with their respective rates of missing data and corresponding numbers from respective guideline’s original study.

**Table 2:** Parameters involved in guideline comparison and corresponding available data from respective guideline’s original study

Parameter	Category	*N*	Missing (%)	BIG [13]	mTBI RS [15]
GCS^a^			n/a		
	15 (%)	435/538 (80.9)		n/a	976/1694 (57.6)
	14 (%)	103/538 (19.1)		n/a	533/1694 (31.5)
Normal first neuro exam	Yes (%)	378/487 (77.6)	51 (9.5)	1049/1232 (85.1)^b^	1377/1610 (85.5)
Intoxicated	Yes (%)	100/538 (18.6)	0 (0)	301/1232 (24.4)^b^	494/1661 (29.7)
Oral blood thinners			0 (0)		
	Anticoagulation (%)	110/538 (20.4)		31/1232 (2.5)^b^	155/1699 (9.1)
	Antiplatelets (%)	148/538 (27.5)		166/1232 (13.5)^b, c^	294/1699 (17.3)
	Any (%)	252/538 (46.8)		197/1232 (16)^b, c^	441/1699 (26.0)
Number of injuries on CT			n/a		
	1 (%)	263/538 (48.9)		n/a	824/1699 (48.5)
	2 (%)	171/538 (31.8)		n/a	400/1699 (23.6)
	3 (%)	69/538 (12.8)		n/a	217/1699 (12.7)
	4 (%)	27/538 (5.0)		n/a	142/1699 (8.4)
	≥ 5 (%)	8/538 (1.5)		n/a	116/1699 (6.8)
Comment on the presence of midline shift^d^	Yes (%)	196/514 (38.1)	n/a	n/a	n/a
Midline shift on initial CT^d^	Yes (%)	95/514 (18.5)	n/a	n/a	159/1699 (9.4%)
Comment on hemorrhage size^d^	Yes (%)	352/514 (68.5)	n/a	n/a	n/a
Hemorrhage under 5 mm^d, e^	Yes (%)	82/514 (16)	n/a	n/a	208/1699 (12.2)
Any skull fracture	Yes (%)	119/538 (22.1)	n/a	544/1232 (44.2)^b, c^	189/1699 (11.1)^c^
ISS ≥ 3 (excluding head)	Yes (%)	112/536 (20.9)	2 (0.4)	n/a	n/a
ISS (excluding head)	Mean (SD)	1.8 (4.1)	2 (0.4)	n/a	5.2 (5.2)

*BIG* brain injury guidelines, *CT* computerized tomography, *GCS* Glasgow Coma Scale, *mTBI* *RS* Mild TBI Risk Score, *ISS* Injury Severity Score, *SD* standard deviation

^a^Converted from the Reaction Level Scale

^b^Estimated using percentages and cohort size from the original study

^c^Assuming patients were only treated with one medication/had one injury

^d^Patients with isolated fractures were not included in these parameters

^e^Either through written in or interpreted from radiology report

The BIG had a sensitivity of 100% (CI 98.1–100%) and a specificity of 2.3% (CI 1–4.5%). The mTBI RS had a sensitivity of 100% (CI 98.1–100%) and a specificity of 2.9% (CI 1.4–5.2%). These results are displayed in Table 3 along with corresponding numbers from respective guideline’s original study.

**Table 3:** Sensitivity and specificity for BIG and HSC DR and corresponding numbers from respective guideline’s original study

	Sens (95% CI)	Spec (95% CI)	*N* disc (%)	Joseph et al. [13]			Marincowitz et al. [15]		
				Sens (95% CI)^a^	Spec (95% CI)^a^	*N* disc (%)	Sens (95% CI)	Spec (95% CI)	*N* disc (%)
BIG	100% (98.1–100)	2.3% (1.0–4.5)	8 (1.5)	100% (97.7–100)	11.28% (9.5–13.3)	121 (9.8)	99.5% (98.5–99.9)	4.8% (3.7–6.3)	57 (3.6)
mTBI RS	100% (98.1–100)	2.9% (1.4–5.2)	10 (1.9)	n/a	n/a	n/a	99.5% (98.5–99.9)	7.4% (6.0–9.1)	87 (5.5)

*BIG* brain injury guidelines, *CI* confidence interval, *mTBI* *RS* Mild TBI Risk Score, *N* *Disc* number of patients eligible for discharge, *Sens* Sensitivity, *Spec* specificity

^a^Based on neurosurgical interventions alone

One-hundred and twenty-six patients (23.4%) had neither the size of their intracranial hemorrhage mentioned in the radiology report nor had a hemorrhage that could be interpreted as less than 5 mm from the radiology report. Assuming all patients had a hemorrhage less than 5 mm, an additional 28 patients would have been eligible for discharge by either BIG (15), mTBI RS (20) or both (7).

Compared to the study by Marincowitz et al. [15], 58.3% (1.5% vs. 3.6%) fewer patients could have been discharged according to the BIG (*P* = 0.0148) and 65% (1.9% vs. 5.5%) fewer patients could have been discharged according to the mTBI RS (*P* < 0.001). Compared to the study by Joseph et al. [13], 84.7% (1.5% vs. 9.8%) fewer patients could have been discharged according to the BIG (*P* < 0.001).

In all cases where no level of consciousness was formally documented in the note from the emergency department (217/538, 40.3%), the level of consciousness could be interpreted from the text in the note. Of these cases, 174 were interpreted as RLS 1/GCS 15 (80.2%) and 43 (19.8%) as RLS 2/GCS 14.

In the second analysis that included patients with RLS 3, an additional 13 patients could be included. Of these 13, none was eligible for discharge according to either the mTBI RS or the BIG.

## Discussion

This study is the first comparative evaluation of two international guidelines regarding the possible discharge of mTBI patients with ICIs directly from the emergency department in a Scandinavian population. This is a large validation cohort of over 500 patients with 188 events, which matches previous sample size recommendations when validating prognostic models [17, 18]. The population in this study is demographically diverse, and the area from which it derived consists of urban and rural areas. This should make the cohort comparable to other similar cohorts in Sweden.

Neither guideline would have discharged a patient who would have deteriorated within 30 days. Application of either guideline would have led to a small but safe reduction of hospital admissions in this population. No significant difference in sensitivity, specificity, or proportion of patients eligible for discharge could be identified between the two guidelines.

Managing mTBI patients without a neurosurgical consultation is a subject that has been studied for some time and proven safe in selected cases. Huynh et al. found that patients with a GCS of 15 and either a solitary contusion less than 5 mm or a subdural hematoma less than 4 mm could safely be managed without a neurosurgical consultation [19]. In their comparative study of patients with mTBI, who were managed either with or without a neurosurgical consultation, Joseph et al. showed that the patients managed with a consultation had higher rates of repeat CT scans and admission to the ICU but not a higher rate of neurosurgical intervention, in-hospital mortality, or readmissions within 30 days [20]. Lewis et al. concluded that routine neurosurgical consultation seemed unnecessary and rarely changed the management plan, even in patients with pre-injury antiplatelet/anticoagulation or intoxication [21].

The specificity and proportion of patients eligible for discharge for both guidelines were lower when compared with their respective original studies. This might, in part, be due to the high levels of missing data in radiological measurements of intracranial hemorrhages. The researchers tried to circumvent this problem by subjectively determining if the hemorrhage was under 5 mm based on how the hemorrhage was described in the radiology report. Despite this effort, only 22.2% of the incomplete records could be completed. Interpretation of the radiology reports was made very conservatively. If there was any doubt whether the hemorrhage was over 4 mm, it was interpreted as such. It is, therefore, possible that a subset of these patients had hemorrhages under 5 mm and would, therefore, have been eligible for discharge based on both guidelines.

The radiologist is an invaluable resource for the emergency care provider, with his/her superior image interpretation. However, the lack of consensus on reporting terminology might lead to the loss of clinically relevant information [22, 23]. Detailed radiology reports in a tabulated format seem to be preferred over prose when non-radiologists are asked. Reports that follow a structured format are perceived as more comprehensible and with better content [24–26].

Suppose all patients who had neither their largest hemorrhage measurement provided nor a hemorrhage that could be interpreted as less than 5 mm had hemorrhages under 5 mm. In that case, an additional 15 patients could have been discharged by the BIG and 20 by the mTBI RS. Using these theoretical numbers, the discharge rates (BIG: 3.9% and mTBI RS: 5%) and specificities (BIG: 6.3% and mTBI RS: 8.3%) for both guidelines were more similar to those received by Marincowitz et al. in their study. However, it is unlikely that all these patients actually had hemorrhages under 5 mm. The lack of precise measurements is thus likely not the only cause for the observed difference in sensitivity and specificity between the original studies and the present study.

The cohorts were different in many aspects between all three studies. The patients in this study were significantly older than either original study. Almost half were treated with anticoagulants or antiplatelets, almost twice the proportion in the mTBI RS study and almost triple the proportion in the BIG study.

The patients in this study were seemingly less severely injured than those in the mTBI RS study when looking at GCS scores and ISS scores. On the other hand, the patients had more skull fractures, more neurological deficits on the initial exam, a higher proportion of midline shift present and deteriorated to a greater extent. These factors, including the use of anticoagulants/antiplatelets, are all reasons for being admitted by either guideline, subsequently reducing the proportion of patients eligible for discharge.

The above-mentioned factors that potentially drive the higher admittance rate can also contribute to the lower specificities through a greater admission rate of patients that do not deteriorate. The more advanced age of the patients in this study is potentially an important factor. Older patients are more likely to have baseline neurological deficits that can be hard to distinguish from new ones on initial examination. The higher rate of cerebral atrophy amongst older patients can also make them more resilient to the mass effect of an intracranial hemorrhage, and thus less prone to develop injuries requiring surgical evacuation. Older patients are more likely to be treated with anticoagulants or antiplatelets and this has shown only a moderately elevated odds ratio for deterioration [15]. This could lead to many patients being admitted, but relatively few that will deteriorate.

The significant differences between the current and previous study populations, along with missing information in radiology reports, can possibly explain the inferior specificities and proportions of patients eligible for discharge by both guidelines in this cohort. However, the exact individual contribution of either of these factors cannot be investigated in the current study.

To have a guideline that helps managing these seemingly well patients with potentially deadly injuries would have been much appreciated by many physicians in the emergency department. At the current moment, neither BIG nor mTBI RS seem to provide much value to the clinical management in the current or similar settings. If their usability and true potential to affect care are to be studied further, precise measurements of ICIs need to be routinely provided.

## Strengths and limitations

One strength of the study is the case identification through ICD-10 codes, which ensured that the researchers would not miss any patient. The data were collected by a single, trained person with presumably less variance in interpretation than if multiple reviewers were used. The data collector had frequent contacts with other team members when necessary for a discussion about the data collection. A detailed control document was developed prior to starting the data collection, extensively defining how ambiguous data should be interpreted.

The RLS scale is the preferred way of measuring the level of consciousness at the researchers’ institution, and the conversions between RLS and GCS are sometimes tricky. This is the reason for choosing to exclude patients belonging to the RLS 3 category (corresponding GCS values would be between 9 and 13) since conversion attempt would introduce too much uncertainty. The second analysis that included patients from the RLS 3 category only marginally increased the cohort size and did not increase the number of patients who could have been discharged based on either guideline. This makes the researchers confident to state that limiting themselves to patients with GCS 14–15 did not affect the end result or the conclusion.

The proportion of patients with no formal documentation of their level of consciousness was almost exclusively interpreted as being fully conscious. A brief description of a patient’s general appearance is often written in the note from the emergency department. When the patient is described as unaffected and alert, physicians might be less prone to formally document the level of consciousness since they believe that the information is already provided.

The choice to interpret the unmentioned findings as their absence was made based on the assumption that admitting and discharging physicians prefer to be concise and not regularly neglect negative findings. Possibly, some patients presented positive findings that were missed because the treating physician failed to either investigate these findings or document them. The data could not be deemed missing at random; therefore, multiple imputations to manage missing data would potentially introduce bias. Furthermore, the parameters used for determining if a patient would have been eligible for discharge are almost exclusively findings that can be objectively verified in the patient’s medical record.

## Conclusion

There was no difference between the two guidelines in sensitivity, specificity, or proportion of the cohort eligible for discharge. Specificity and proportion of cohort eligible for discharge were lower than each guideline’s original study. At present, neither guideline can be recommended for implementation in the current or similar settings.

## Supplementary Information

Below is the link to the electronic supplementary material.

Supplementary file1 (PDF 98 kb)

Supplementary file2 (PDF 176 kb)

## Data Availability

The source data are available from the corresponding author upon reasonable request.
