# Supplementary material for: Comparison of the predictive value of two international guidelines for safe discharge of patients with mild traumatic brain injuries and associated intracranial pathology
Source: Eur J Trauma Emerg Surg. 2021 Dec 3;48(6):4489–97. doi: 10.1007/s00068-021-01842-6 (PMC9712145; doi:10.1007/s00068-021-01842-6)
Supplement: Supplementary file 1 — Supplementary file1 (PDF 98 kb) [file 68_2021_1842_MOESM1_ESM.pdf]

Supplementary material 2: Coding Template

Title:

Performance of the Brain Injury Guidelines and the Mild Traumatic Brain Injury Risk Score in a Scandinavian population – A Retrospective Chart Review

Journal:

European Journal of Trauma and Emergency Surgery

Authors:

Sebastian Vestlund **(Corresponding author)**
Faculty of Medicine – Department of Clinical Sciences,
Lund University, Sweden
Sebastian.Vestlund@med.lu.se
+46708244127

Sebastian Tryggmo
Faculty of Medicine,
Lund University, Sweden

Tomas Vedin
Faculty of Medicine – Department of Clinical Sciences
Lund University, Sweden

Per-Anders Larsson
Faculty of Medicine – Department of Clinical Sciences
Lund University, Sweden

Marcus Edelhamre
Faculty of Medicine – Department of Clinical Sciences
Lund University, Sweden

1. **SEX - Gender**

0 = Female
1 = Male

2. **AGE - Age**

Fulfilled years

3. **ADM_DATE - Admission date**

YYYY-MM-DD.

4. **ADM_TIME - Admission time**

HH: MM

5. **DISC_DATE - Discharge date**

YYYY-MM-DD

6. **DISC_TIME - Discharge time**

HH: MM

7. **n_DAYS - Number of days in hospital**

Number of full days.

8. **n_HOURS - In less than a day, number of hours?**

HH: MM
88 = Not relevant

9. **EXCL - Excluded?**

0 = No
1 = Yes

10. **EXCL_WHY - If excluded, why?**

0 = Not excluded
1 = Incorrectly registered as intracranial injury, alternatively non-traumatic injury
2 = Double
3 = Re-admission for already included injury
4 = Transferred from another hospital
5 = Journal with extended confidentiality
6 = Transferred to another region before discharge
7 = Died after last registered care opportunity
8 = Initial CT missing
9 = Patient from another region / from abroad
10 = Penetrating injury

11. **COAGPATH - Known congenital coagulation disorder?**

0 = No
1 = Yes

If nothing is stated in the record for the current care session, this is interpreted as 0.

12. TIME_TRAUMA - Time from trauma to emergency room?

1 = 0-6 h
2 = 6-12 h
3 = 12-24
4 = < 24 h
5 = > 24 h
6 = > a week
7 = > a month
99 = Data missing

If the time cannot be assessed with certainty, state 99. If it appears that the injury occurred within a day but can not be determined closer than that, alternative 4.

13. MECHANISM - Trauma mechanism

1 = Fall in the same level(from sitting or standing)
2 = Fall from height
3 = Assault
4 = Traffic accident
5 = Sports injury
6 = Other
7 = Unknown
99 = Not stated in journal

14. AMNESIA - Amnesia after the injury?

0 = No
1 = Yes
2 = Not mentioned in the journal

15. AMNESIA_TYPE - If amnesia, what type?

1 = Antegrade
2 = Retrograde
3 = Patient can not characterize amnesia as retrograde or antegrade
4 = Combined antegrade and retrograde
88 = Not relevant
99 = Data missing

16. AMNESIA_DURATION - Amnesia duration?

1 = Under 30 min
2 = Over 30 min
3 = Unknown duration
4 = Not stated in journal
88 = Not relevant

17. LOC - Loss of consciousness?

0 = No
1 = Yes
2 = Not mentioned in journal
3 = Uncertain

99 = Data missing

18. LOC_DURATION - If loss of consciousness, duration?

1 = "Momentarily”
2 = <1 min
3 => 1 min <5 min
4 => 5 min
5 = Patient does not know
6 = Unconscious on arrival
88 = Not relevant
99 = Data missing / no assessment of duration stated

19. INTOX - Intoxicated ?

0 = Not intoxicated
1 = Alcohol
2 = Cannabis
3 = Central stimulants (Cocaine, Amphetamine, Methamphetamine, Methylphenidate, LSD, Ecstacy)
4 = Opiates (Heroin, Morphine, Oxycodone, Codeine, Tramadol)
5 = Benzodiazepines (Xanor, Iktorivil, Storivol, Storivol Oxascand)
6 = Unknown substance
7 = Pat can not report
8 = Other
9 = Mixed intoxication
99 = No data

History of intake of any substance, laboratory findings indicating intake, alcohol odor or other abnormalities that are primarily interpreted as intoxication should be stated as intoxicated. "Unknown substance" is used if the type of substance is not specified. "Not intoxicated" is stated if the journal does not comment on the subject.

20. ALC_ABUSE - Suspicion of chronic alcoholism based on the note?

0 = No
1 = Yes

21. SEIZURE_PREHOSP - Seizure before arriving at hospital?

0 = No
1 = Yes
2 = Not mentioned
3 = Possible

22. n_SEIZURE_PREHOSP - If seizures, number of episodes?

Figure.
88 = Not relevant
99 = Cannot be determined with certainty

23. SEIZURE_ED - Seizures in the emergency department?

0 = No
1 = Yes

2 = Not mentioned journal

24. n_SEIZURE_ED - About seizures in hospital, number of episodes?

Figure.
88 = Not applicable

25. VOMIT_PREHOSP - Vomiting before arrival at the hospital?

0 = No
1 = Yes
2 = Not mentioned

26. n_VOMIT_PREHOSP - About vomiting, number of episodes?

Figure.
88 = Not relevant
99 = Can not be determined with certainty

27. VOMIT_ED - Vomiting in the emergency department?

0 = No
1 = Yes
2 = Not mentioned

28. n_VOMIT_ED - About vomiting in hospital, number of episodes?

Figure.
0 = Number not specified.
88 = Not relevant
99 = Cannot be determined with certainty

29. HEADACHE - Headache after injury?

0 = No
1 = Yes
2 = Not mentioned

30. DOC_RLS - Formal documentation of RLS in the emergency department note?

0 = No
1 = Yes

31. DOC_RLS_LEVEL - If yes, RLS level at the first listing?

1–8.
88 = Not relevant

32. INT_RLS - If no, possible to determine RLS from the note?

0 = No
1 = Yes

33. INT_RLS_LEVEL - If yes, RLS level?

1–8.
88 = Not relevant

34. DOC_GCS - Formal documentation of GCS in the emergency department?

0 = No
1 = Yes

35. DOC_GCS_LEVEL - If yes, GCS level at the first listing?

3–15.
88 = Not relevant

36. INT_GCS - If no, possible to determine GCS 15 from the note?

0 = No
1 = Yes

37. INT_GCS_LEVEL - If yes, GCS level?

3–15.
88 = Not relevant

38. NORM_NEURO_EX - Normal neurological examination in the emergency department?

0 = No
1 = Yes
2 = Cannot be valued from the note due to. insufficient documentation.
3 = No neurological examination documented.

39. EQ_PUPILS - Pupils of the same size at the first examination?

0 = No
1 = Yes
2 = Not stated in the medical record
3 = Cannot be evaluated based on the note (eg one-eyed, swollen)

40. REACT_PUPILS - Both pupils are light-reactive at the first examination?

0 = No
1 = Yes
2 = Not stated in journal
3 = Cannot be evaluated

41. SIGNS_SKULLFX - Signs of a skull fracture?

0 = No signs of skull fracture
1 = Palpable level difference on examination
2 = Palpable fluctuation in the skull
3 = Boggy hematoma
4 = Retroauricular hematomas (Battle sign)
5 = Hematotympanon
6 = Clear secretion from ear or nose
7 = Multiple signs
8 = Not commented in journal

If no note mentions the presence or absence of external injuries to the head, select 8.

42. CARDVASC_DIS - Cardiovascular disease?

0 = No
1 = Yes

- Past myocardial infarction
- Heart failure
- Previous TIA / Stroke
- Peripheral vascular disease
- Completed reperfusion surgery (CABG, PCI, PTA)
- Heart transplant

43. DIABETES_MELL - Diabetes mellitus?

0 = No
1 = Yes

44. PULM_DIS - Lung disease?

0 = No
1 = Yes

- COPD
- Pulmonary fibrosis
- Severe asthma

45. ESRD - Kidney failure with dialysis / Chronic kidney failure with eGFR <30?

0 = No
1 = Yes

46. LIVER_DIS - Liver disease?

0 = No
1 = Yes

- Liver cirrhosis
- Other liver diseases with affected liver enzymes

47. NEURO_DIS - Neurological disease?

0 = No
1 = Yes

- Multiple sclerosis
- Amyotrophic lateral sclerosis
- Epilepsy
- Guillan-Barré syndrome
- Myasthenia gravis
- Parkinson's

48. DEMENTIA - Dementia?

0 = No
1 = Yes

Regardless of degree or cause.

49. PSYCH_DIS - Psychiatric illness?

0 = No
1 = Yes

- Severe depression
- Bipolar disorder
- Chronic psychotic disorders
- Dementia with delusions that have required inpatient psychiatric care for the past 5 years

50. ANTICOAG - Anticoagulants?

0 = No
1 = Waran (Warfarin)
2 = Eliquis (Apixaban)
3 = Pradaxa (Dabigatran)
4 = Xarelto (Rivaroxaban)
5 = Lixiana (Edoxaban)
6 = LMWH (Low molecular weight heparin)
99 = Data missing

If the journal takes up the point ”current medication ”without mentioning the above preparation is stated 0. Alternatively, 0 is chosen even when“ current medication ”is not commented on and where the patient does not, according to the medical record, meet the known indication for anticoagulation, eg young and healthy patients. In other cases where no preparation is reported, state 99.

51. TRCINHIB - Platelet inhibitor?

0 = No
1 = Thrombyl (ASA)
2 = Plavix (Clopidogrel)
3 = Brilique (Ticagrelor)
4 = Efient (Prasugrel)
5 = Persantin (Dipyramidol)
6 = PLETAL (Cilostazol)
99 =No data

52. DBLTRCINHIB - Dual Platelet Inhibition?

See list 13.

53. CFS_GRADE - Clinical frailty scale-degree?

0 = <50 years
Otherwise graduation 1-9 according to the scale below.

1. **Very vital -** individuals who are strong, active, energetic, and motivated. They often exercise regularly. They belong to those who are in the best condition for their age.
2. **Vital -** individuals who have no disease symptoms but who are in worse shape than individuals in category 1. They often train or are occasionally very active, e.g. depending on the season.

3. **Doing well -** individuals whose medical problems are well controlled, but who are not regularly active in addition to regular walks.
4. **Vulnerable -** does not depend on the help of others in everyday life, but often has symptoms that limit their activities. A common complaint is that they are limited ("slowed down") and/or get tired during the day.
5. **Mild frailty -** these individuals are often noticeably slower and need help with complex IADL (Instrumental Activities of Daily Living) activities (finances, transportation, heavy housework, medication). Mild fragility generally impairs the ability to shop and go out on your own, cook, and do housework.
6. **Moderately frail -** individuals who need help with all outdoor activities and housework**.** Indoors, often have problems with stairs, need help with washing, and may need minimal help (encouragement, support) to get dressed.
7. **Severely frail -** is completely dependent on others for personal self-care regardless of cause (physical or cognitive). Nevertheless, they appear to be stable and without a high risk of dying (within about 6 months).
8. **Very seriously frail -** completely addicted, nearing the end of life. They can generally not recover even from a mild illness.
9. **Terminally ill -** nearing the end of life. This category includes individuals with an expected remaining life expectancy of less than 6 months with no other obvious signs of fragility.

54. DEATH_30D - Death within 30 days from primary visit noted in patient record?

0 = No
1 = Yes

55. DEATH_ADM - Death during the current care session?

0 = No
1 = Yes

56. DEATH_24H - Died within 24 hours of primary visit?

0 = No
1 = Yes

57. DEATH_ICI - Death secondary to traumatic intracranial injury?

0 = No
1 = Yes
88 = Not relevant

58. DEATH_ICI_DoI - Directly or indirectly caused by traumatic intracranial injury?

1 = Direct
2 = Indirect
88 = Not relevant

59. DEATH_DATE - Date of death

YYYY-MM-DD
88 = Not relevant

60. NEURO_INT_30D - Neurosurgical operation or procedure within 30 days of primary visit?

0 = No
1 = Yes

61. NEURO_INT_ADM - Neurosurgical intervention during the current care session?

0 = No
1 = Yes

62. NEURO_INT_24H - Neurosurgical intervention within 24 hours?

0 = No
1 = Yes
88 = Not relevant

63. NEURO_INT_TYPE - Which intervention (s)?

1 = Craniotomy
2 = Burrhole
3 = Intracranial pressure measurement
4 = Mannitol
5 = Hyperventilation
6 = Hypertonic saline
7 = Intravenous antibiotics due to skull base fracture
8 = Other
9 = Multiple interventions
88 = Not relevant

64. NEURO_INT_DATE - Date of the first intervention

YYYY-MM-DD
88 = Not applicable

65. SEIZURE_30D - Documented seizures within 30 days of primary visit?

0 = No
1 = Yes

66. SEIZURE_ADM - Cramps during the current care session?

0 = No
1 = Yes

67. SEIZURE_24H - Cramps within 24 hours of arrival?

0 = No
1 = Yes
88 = Not relevant

68. SEIZURE_DATE - Date of first seizure

YYYY-MM-DD
88 = Not relevant

69. DEC_CONC_30D - Decreased consciousness within 30 days of primary visit?

0 = No
1 = Yes

Defined as a decrease of 1 point in RLS or 2 points in GCS.

70. DEC_CONC_ADM - Decreased consciousness during the current care session?

0 = No
1 = Yes

71. DEC_CONC_24H - Decreased consciousness within 24 hours?

0 = No
1 = Yes
88 = Not relevant

72. DEC_CONC_DATE - Date of the first episode of decreased consciousness

YYYY-MM-DD
88 = Not relevant

73. ICU_30D - Care in the intensive care unit within 30 days from the primary visit?

0 = No
1 = Yes

74. ICU_ADM - Care in the intensive care unit during the current care session?

0 = No
1 = Yes

75. ICU_24H - Care in the intensive care unit within 24 hours from the primary visit?

0 = No
1 = Yes
88 = Not relevant

76. ICU_ICI - Care in the intensive care unit due to intracranial injury?

0 = No
1 = Yes
2 = Multiple care episodes due to ICI
3 = Multiple care episodes due to ICI + other cause
4 = Multiple care episodes due to other cause
88 = Not relevant

77. ICU_DATE - Date of enrollment in the intensive care unit

YYYY-MM-DD
88 = Not relevant

78. INTUB_30D - Acutely intubated within 30 days from the primary visit due to intracranial injury?

0 = No
1 = Yes

79. INTUB_ADM - Acutely intubated during the current care session?

0 = No
1 = Yes

80. INTUB_24H - Acute intubated within 24 hours from primary visit due to. intracranial injury?

0 = No
1 = Yes
88 = Not relevant

81. INTUB_DATE - Date of acute intubation

YYYY-MM-DD
88 = Not applicable

82. READMIT_30D - Involuntary re-admission to the hospital within 30 days of primary visit linked to the current injury?

0 = No
1 = Yes

83. READMIT_DATE - Date for re-admission

YYYY-MM-DD
88 = Not applicable

84. DISC_REH_TBI - Discharged to rehab facility due to TBI?

0 = No
1 = Yes

85. BP_SYST - First noted systolic blood pressure

mmHg
0 = No value during the first day

86. BP_DIAST - First noted diastolic blood pressure

mmHg
0 = No value first day

87. PULSE - First recorded heart rate

Stroke / minute
0 = No value first day

88. 02_SAT - First noted oxygen saturation

percentage
0 = No value first day

89. RESP_FRQ - First noted respiratory rate

breaths per minute
0 = No value first day

90. HB - First noted Hb values in the ED

g / L

0 = Not sampled

91. TRC - First noted platelet count in the ED

10^9/ L
0 = Not sampled

92. GLU - First noted blood sugar value at the emergency department

mmol / L
0 = Not sampled

93. PK_INR - First noted PK (INR) value at the emergency department

Number
0 = Not sampled

94. APT_T - First noted APT time in the emergency room

Seconds
0 = Not sampled

95. CT_INJ_1 - Injury on CT # 1

1 = Subdural hemorrhage, acute
2 = Subdural haemorrhage, subacute / chronic
3 = Epidural hemorrhage
4 = Subarachnoidal hemorrhage
5 = Parenchymal hemorrhage
6 = Contusion hemorrhage
7 = Other intracranial hemorrhage
8 = Signs of DAI injuries
9 = Signs of cerebral edema
10 = Skull base fracture
11 = Crush fracture without impression
12 = Crush fracture with an impression
13 = Linear fracture without impression
14 = Linear fracture with an impression
15 = Other skull fracture
16 = Hemorrhage difficult to characterize

96. CT_INJ_2 - Injury to CT # 2

See list 95.

97. CT_INJ_3 - Injury to CT # 3

See list 95.

98. CT_INJ_4 - Injury to CT # 4

See 95

99. CT_INJ_5 - Injury to CT # 5

See 95

100. n_CT_INJ - Number of isolated injuries on CT examination?

Number

101. OLD_CT_INJ - Older bleeding on CT?

0 = No
1 = Yes

102. MID_SHIFT_WRITTEN - Centerline Offset / Mass Power?

0 = No
1 = Yes
2 = Not mentioned

103. MID_SHIFT_MM - If yes, number of millimeters?

mm
88 = Not relevant
99 = Dimensions not specified in journal

104. LARGEST_BLEED_WRITTEN - Written comment on the size of the largest intracranial hemorrhage?

0 = No
1 = Yes

105. LARGEST_BLEED_MM - If yes, number of millimeters?

mm
0 = If occurrence but size not specified
88 = Not relevant

106. <5MM_WRITTEN - Written report raises suspicion of bleeding less than 5 mm?

0 = No
1 = Yes

107. ATROPHY - Comment regarding the presence of cerebral atrophy in statement?

0 = No
1 = Yes

108. REP_CT - At least 1 repeated CT Brain during the current care session?

0 = No
1 = Yes

109. REP_CT_WHY - Cause of Repeated CT?

1 = Decreased consciousness
2 = Increasing headache
3 = New neurological abnormality
4 = Vomiting
5 = Other
6 = Reason not clear
7 = Supplementary examination
88 = Not applicable

110. REP_CT_WORSE - Deterioration on CT?

0 = No change
1 = Deterioration of existing damage
2 = New damage
3 = New damage + deterioration of existing damage
88 = Not applicable

111. AIS_FACE - Maximum AIS value on the face.

0–6

Wound injuries, hematomas, and abrasions are excluded from this grading and are assessed as 0. If multiple injuries within the same region, the one with the highest value is selected.

112. AIS_NECK - Maximum AIS value on the neck

See 111.

113. AIS_THX - Maximum AIS value from the chest

See 111

114. AIS_ABD - Maximum AIS value from the abdomen

See 111.

115. AIS_SPINE - Maximum AIS values from the spine

See 111.

116. AIS_W- SPINE - Maximum AIS value from the entire spine as a unit

See 111.

117. AIS_C-SPINE - Maximum AIS value from the cervical spine

See 111.

118. AIS_T-SPINE - Maximum AIS value from the thoracic spine

See 111.

119. AIS_L-SPINE - Maximum AIS value from the lumbar spine

See 111.

120. AIS_U-EXT - Maximum AIS value from the upper extremities

See 111.

121. AIS_L-EXT - Maximum AIS value from the lower extremities

See 111.
