# Supplementary material for: Comparison of the predictive value of two international guidelines for safe discharge of patients with mild traumatic brain injuries and associated intracranial pathology
Source: Eur J Trauma Emerg Surg. 2021 Dec 3;48(6):4489–97. doi: 10.1007/s00068-021-01842-6 (PMC9712145; doi:10.1007/s00068-021-01842-6)
Supplement: Supplementary file 2 — Supplementary file2 (PDF 176 kb) [file 68_2021_1842_MOESM2_ESM.pdf]

# <u>Supplementary material 1: Included ICD-10 codes</u>

**Title:**

Performance of the Brain Injury Guidelines and the Mild Traumatic Brain Injury Risk Score in a Scandinavian population – A Retrospective Chart Review

**Journal:**

European Journal of Trauma and Emergency Surgery

**Authors:**

Sebastian Vestlund **(Corresponding author)**
Faculty of Medicine – Department of Clinical Sciences,
Lund University, Sweden
Sebastian.Vestlund@med.lu.se
+46708244127

Sebastian Tryggmo
Faculty of Medicine,
Lund University, Sweden

Tomas Vedin
Faculty of Medicine – Department of Clinical Sciences
Lund University, Sweden

Per-Anders Larsson
Faculty of Medicine – Department of Clinical Sciences
Lund University, Sweden

Marcus Edelhamre
Faculty of Medicine – Department of Clinical Sciences
Lund University, Sweden

**<u>Included ICD-10 codes</u>**

**S02.0 –** Fracture of Vault of Skull

**S02.1 –** Fracture of Base of Skull

**S02.7 –** Multiple Fractures Involving Skull and Facial Bones

**S02.8 –** Fractures of Other Skull and Facial Bones

**S02.9 –** Fracture of Skull and Facial Bones, Part Unspecified

**S06.1 –** Traumatic Cerebral Edema

**S06.2 –** Diffuse Traumatic Brain Injury

**S06.3 –** Focal Traumatic Brain Injury

**S06.4 –** Epidural Hematoma

**S06.5 –** Traumatic Subdural Hematoma

**S06.6 –** Traumatic Subarachnoid Hemorrhage

**S06.7 –** Intracranial Injury with Prolonged Coma

**S06.8 –** Other Specified Intracranial Injuries

**S06.9 –** Unspecified Intracranial Injury
